# Should vegans have children? A response to Räsänen

**DOI:** 10.1007/s11017-024-09664-4

**Published:** 2024-05-14

**Authors:** Louis Austin-Eames

**Affiliations:** Philosophy, School of Humanities, University of Glasgow, Glasgow, Scotland

**Keywords:** Veganism, Anti-natalism, Unnecessary suffering, Utilitarian, Räsänen

## Abstract

Joona Räsänen argues that vegans ought to be anti-natalists and therefore abstain from having children. More precisely, Räsänen claims that vegans who accept a utilitarian or rights-based argument for veganism, ought to, by parity of reasoning, accept an analogous argument for anti-natalism. In this paper, I argue that the reasons vegans have for refraining from purchasing animal products do not commit them to abstaining from having children. I provide novel arguments to the following conclusion: while there is good reason to believe that factory farming results in a net disutility and involves treating non-human animals as mere means, there is not good reason to believe that having children results in a net disutility or involves treating the children as mere means. Subsequently, I respond to what I take to be Räsänen’s underlying reasoning—that vegans are committed to abstaining from other practices which cause unnecessary suffering. I respond by arguing that this is plausibly false as various practices which cause unnecessary suffering are likely permissible, whereas factory farming is not.

## Introduction

Räsänen [1] argues that vegans ought to be anti-natalists and therefore abstain from having children. Before arguing against Räsänen’s position, I will clarify some of the terminology that I will use throughout the paper. I will use the term “veganism”, much like Räsänen [1] has, to denote the view that it is wrong to use animals in such a way that causes them unnecessary suffering.^1^ This includes eating meat and animal products, such as milk and eggs, and purchasing leather, suede, wool, etc.^2^ I will also be using the term “anti-natalism”, much like Räsänen [1] has, to denote the view that it is wrong to procreate because doing so harms the person that is brought into existence. This understanding of anti-natalism aligns with the way Benatar [3] uses the term, in that the motivation is to thwart the suffering of the potential child by not bringing them into existence in the first place.^3^

Having cleared up the terminology, I can now turn to Räsänen’s view. Roughly speaking, Räsänen argues that vegans should abstain from having children because it causes unnecessary suffering. This is because it is unnecessary to have children and the prospective children would inevitably suffer throughout their lives [1]. Since a primary motivation for being vegan, according to Räsänen, is to prevent unnecessary suffering (i.e., to the animals who suffer in factory farming), vegans also ought to abstain from having children in virtue of the suffering the prospective children would experience throughout their lives. More specifically, Räsänen provides both utilitarian and rights-based arguments for veganism. Subsequently, he provides analogous utilitarian and rights-based arguments for anti-natalism, arguing that vegans who accept one or both arguments for veganism, ought to, by parity of reasoning, accept one or both arguments for anti-natalism [1]. Put differently, on pain of contradiction, vegans who accept the arguments for veganism should also accept the analogous argument(s) for anti-natalism.

In this paper, I will attempt to show that it is in fact permissible for vegans to have children. I will do this by rejecting the supposed parity between the arguments for veganism and those for anti-natalism, arguing that vegans need not be anti-natalists. More precisely, I will argue that although all Räsänen’s arguments are valid, only those for veganism are sound. In the second section, I will provide an exposition of the utilitarian arguments for veganism and those for anti-natalism on offer from Räsänen. Thereafter, I will argue that while there is good reason to accept that factory farming results in a net disutility, there is not good reason to accept the claim that having children results in a net disutility. In the third section, I will lay out the rights-based arguments for veganism and anti-natalism. Subsequently, I will argue that while it seems plausible that factory farmed animals are used as mere means, it seems implausible that having children uses said children as mere means. In the fourth section, I will address the overarching theme of the paper, namely that since preventing unnecessary suffering is what motivates many vegans to abstain from supporting factory farming, that they should also abstain from having children given that this also prevents unnecessary suffering.

## Utilitarian arguments

Before examining the utilitarian arguments for veganism and anti-natalism, I want to make it clear that Räsänen [1] is not focusing on whether the arguments are sound. That is, according to Räsänen [1], both veganism and anti-natalism could be false. Rather, he is focusing on the relationship between the arguments for veganism and those for anti-natalism. To this end, I also want to make it clear that in calling into question the soundness of some of the arguments, as I will do shortly, I am not attempting to strawman Räsänen. I will be calling into question the soundness of the arguments for anti-natalism to show that there is an asymmetry between the arguments made for veganism and those made for anti-natalism, such that vegans need not accept the arguments for anti-natalism.

Räsänen’s utilitarian argument for veganism is as follows:

‘P1) If factory farming causes greater suffering and interest-frustration on non-human animals than what humans would experience if they had to abstain from eating meat, then factory farming is immoral.

P2) Factory farming causes greater suffering and interest-frustration on non-human animals than what humans would experience if they had to abstain from eating meat.

C) Factory farming is immoral’ [1, p. 143].

And his utilitarian argument for anti-natalism is as follows:

‘P1) If having children causes greater suffering and interest-frustration on the prospective child than what prospective parents would experience if they had to abstain from having children, then having children is immoral.

P2) Having children causes greater suffering and interest-frustration on the prospective child than what prospective parents would experience if they had to abstain from having children.

C) Having children is immoral’ [1, p. 145].

Räsänen [1] takes it to be the case that many vegans will find something akin to the above utilitarian argument for veganism plausible. Consequently, he contends that vegans should also find the utilitarian argument for anti-natalism plausible and should therefore abstain from having children. After all, Räsänen argues that P1 of the anti-natalist argument is a straightforward case of utilitarian reasoning, whereas P2 seems plausible, in part, because the prospective children would likely suffer more throughout their *entire lives*, than the prospective parents would if they had to live the *last three quarters of their lives* without children [1, p. 145]. The thought behind this latter consideration seems to be that the prospective parents would suffer for a shorter duration if they were to abstain from having children than the prospective children would if they were brought into existence. Thus, it seems that as far as utility is concerned, abstaining from having children is preferable to having them, all else being equal.

I want to offer a two-part response to the utilitarian arguments. I want to start by pointing out that Räsänen’s utilitarian argument for veganism implies a false dichotomy, namely that one must *either* support factory farming *or* be vegan. This is not true, however, as one can both refrain from supporting factory farming *and* not be vegan simultaneously. For example, one could purchase animal products exclusively from humane farms. Hence, Räsänen’s utilitarian argument for veganism is actually an argument against factory farming. That being said, it is worth noting, in defense of Räsänen, that the vast majority (approximately 75—90% globally) of farmed animals are factory farmed [6, 7]. Therefore, although boycotting factory farming does not entail veganism, it does entail abstaining from the vast majority of animal products given the way they are currently produced.^4^ Given the clarification above, I do not think it is misleading to continue to use veganism as Räsänen has, to denote the anti-factory farming position, at least for the purposes of this paper, which is to engage Räsänen on their own terms and attempt to show that vegans need not be anti-natalists.

The second part of my response will be to reconstruct the utilitarian arguments for veganism and anti-natalism as P1 appears to be false in both. Subsequently, I will then argue that even when one reconstructs and bolsters the arguments, only the utilitarian argument for veganism appears to be sound. To start things off, P1 in both arguments is false because under any utilitarian theory, the moral status of an action is determined by the *net* utility, or disutility, generated by the action. Under classic utilitarianism, for example (the kind subscribed to by Jeremy Bentham and John Stuart Mill), one ought to bring about the greatest good, understood in terms of pleasure or wellbeing, to the greatest number of individuals [8–10].

The above arguments do not consider the net utility of veganism and anti-natalism. To illustrate why, consider P1 of the argument for anti-natalism. This premise compares the prospective child’s suffering (if they are brought into existence) with the prospective parent’s suffering (if they were to abstain from having the child). However, it does not take into account the wellbeing the prospective child would experience if brought into existence, nor does it take into account the wellbeing the prospective parents would experience if they were to have the child. To figure out the net utility of having children, relative to abstaining, one must factor in said variables.^5^ This is because even if the prospective child would in fact experience more suffering throughout their life, than the prospective parents would if they abstained from having children, it could nevertheless be the case that having children results in a net utility. In other words, it could well be the case (and I suspect it often is) that the prospective child and the prospective parents both experience sufficient wellbeing to outweigh the suffering.^6^ Likewise, with respect to P1 of the utilitarian argument for veganism, one must also take into account the wellbeing of the non-vegan (which is generated from eating meat and animal products, etc.) and the wellbeing experienced by the animals being factory farmed. With this in mind, I propose the following amended arguments which bypass this problem.

First, here is the amended utilitarian argument for veganism:

P1) If supporting factory farming causes greater suffering and interest-frustration than not supporting factory farming, then supporting factory farming is immoral.

P2) Supporting factory farming causes greater suffering and interest-frustration than not supporting factory farming.

C) Factory farming is immoral.

And second, here is the amended utilitarian argument for anti-natalism:

P1) If having children causes greater suffering and interest-frustration than abstaining from having children, then having children is immoral.

P2) Having children causes greater suffering and interest-frustration than abstaining from having children.

C) Having children is immoral.

With the amended arguments in place, I will now argue that there is good reason to accept that the utilitarian argument for veganism is sound, whereas there is no such reason to accept that the anti-natalist argument is sound. Consequently, I will attempt to show that vegans who accept the utilitarian argument for veganism need not accept the utilitarian argument for anti-natalism.

### Veganism and utility

I want to start by providing my reasoning in favour of P2 of the argument for veganism. Firstly, although the sentience of non-human animals may differ quite drastically from human sentience, it seems uncontentious that the non-human animals who are standardly factory farmed, i.e., cows, pigs, and chickens, are sentient and capable of suffering [11]. Secondly, it seems uncontroversial that factory farms are not conducive to animal wellbeing. On the contrary, it seems that they cause a significant amount of suffering to the billions of animals who are factory farmed each year [12]. This is because factory farmed animals are generally confined to small spaces indoors, such that they are unable to perform normal behaviours such as nesting or foraging. Being restricted to such small spaces can also lead to the animals injuring one another because of frustration and stress. To reduce such injuries, it is common to mutilate the animals by trimming their beaks, docking their tails, and clipping their teeth. Supposedly, this is often carried out with no anaesthetics. Moreover, animals are often selectively bred so that they grow quickly. As a result, they often suffer from broken bones and organ failure [13–15]. In addition to this, the animals are then slaughtered at a young age. Thus, it seems very clear that these practices result in the animals experiencing a great deal of suffering in addition to having their basic interests, such as living freely and avoiding pain, frustrated.

It seems likely that the animal suffering per se is sufficient to render factory farming immoral on utilitarian grounds. That is to say that the animal suffering is plausibly enough to make it such that factory farming results in a net disutility. However, there are additional well-established sources of disutility which are exacerbated by factory farming that I want to address briefly. One of these is the impact of factory farming on climate change and the environment more broadly. For example, animal agriculture seems to account for somewhere between 11 and 18% of greenhouse gases worldwide, which is similar to all forms of transport combined.^7^ Another is the significant amount of water pollution caused by factory farming [19]. These environmental factors also count against factory farming insofar as utility is concerned given the consequences of climate change and pollution. In short, there is significant disutility which results from factory farming.

What about the utility generated by factory farming? As far as the animals are concerned, it is clear that whatever wellbeing they will experience on factory farms will be outweighed by the suffering brought about by the aforementioned practices and stressful living conditions. However, what about the wellbeing (e.g., taste, convenience) humans experience from the consumption of meat and animal products? In response, it is surely mistaken to suppose that the human wellbeing which results from consuming animal products is sufficient to outweigh the disutility generated by factory farming. This is because the factory farmed animals will likely suffer substantially because of the aforementioned practices for years before they are slaughtered. By contrast, the humans who are consuming the animal products will experience a fairly small and fleeting amount of wellbeing when consuming animal products. Moreover, this is assuming that those who consume meat and animal products would not experience an equal or greater amount of wellbeing if they were to switch to a vegan diet and consume the vegan alternatives. It is not clear that one should make such an assumption.

One might object here that I am mistaken for assuming that more suffering results from non-vegan diets than vegan ones. For example, Deckers [20, p. 78] argues that diets which avoid the deliberate killing of animals do not necessarily result in less suffering than diets which involve the deliberate killing of animals for meat consumption. In other words, Deckers contests that vegan diets always result in fewer animal deaths. To this end, Deckers cites Steven Davis [21, 22], who has argued that some vegan diets cause a lot more harm to animals than other diets due to the agricultural practices required for arable farming, such as ploughing [20, p. 78]. Moreover, Deckers [20, p. 78] expands on Davis’ work, arguing that if one considers invertebrate animals, such as earthworms, arable farming kills more animals than Davis estimates thereby increasing the likelihood that vegan diets may cause greater suffering than non-vegan diets.

In response to this consideration, I want to make two points. Firstly, the data regarding animal deaths in plant agriculture is complex and Davis’ estimates have received substantial criticism [23–26]. Matheny [23], for example, criticises Davis for using total, rather than per capita estimates of animal deaths, whereas Fischer and Lamey [26] claim that Davis’ approach of generalizing from mice deaths in grain production, to overall animal death from plant agriculture, is problematic. Therefore, although Deckers highlights important and relevant factors which must be considered when evaluating the harm which results from vegan and non-vegan diets, it seems like as things currently stand, there is not good enough reason to accept Davis’ estimations that vegan diets cause more animal suffering.

Secondly, and I think more importantly, there is a moral asymmetry, in terms of utility, between killing factory farmed animals for food on the one hand, and animal deaths which result from crop production on the other. A diet which involves supporting factory farming, i.e., bringing animals into existence to live short lives which are plausibly characterised by suffering and a premature death, is a great source of disutility which is constitutive of the vast majority of non-vegan diets. Conversely, however, it is not clear that animal deaths from crop production result in a net disutility. That is, it may actually be a good thing, in terms of utility, that these crop deaths occur given that there is reason to believe that the alternative may be worse.

More precisely, as highlighted by Fischer and Lamey [26], among others,^8^ there is good reason to believe that a great number of field animals’ lives are characterised by disutility. Very roughly, this is because most field animals are r-strategists with respect to reproduction. That is, they have as many offspring as possible and do not invest resources in any of them, such that only a minority will go on to reproduce. The consequence of this is that the majority of these animals die of starvation, get eaten alive by predators, have debilitating genetic abnormalities, or succumb to the elements. Hence, even if one granted the very controversial claim that more animals die as a result of a vegan diet, there is some reason to believe that this may be preferable to them continuing lives characterised by disutility before dying anyway. It is worth highlighting that this problem does not occur for factory farmed animals, as such animals would not be brought into existence in the first place if there was no longer demand for such animal products.

One final consideration worth mentioning is how veganism relates to human health. It could be objected that the consumption of meat and animal products thwarts a major source of disutility, namely health complications such as nutritional deficiencies which result from a vegan diet. In response, a detailed discussion regarding the intricacies of human health on a vegan diet are out with the scope of this paper. However, I will say that such a concern seems unwarranted in virtue of various major bodies of nutritional experts, such as the American Dietetic Association, concluding that a well-planned vegetarian diet (including a fully vegan diet) is healthful, nutritionally adequate, may provide health benefits in the prevention and treatment of certain diseases, and is appropriate for all stages of life.^9^ In summary, the animal suffering and environmental damage caused by factory farming provides good reason to go vegan on utilitarian grounds, whereas the utility generated by factory farming pales in comparison.

### Anti-natalism and utility

With respect to anti-natalism, I will argue that there is not good reason to accept that abstaining from having children results in a net utility.^10^ Before proceeding, however, I want to draw attention to a difficulty which arises when thinking about the aggregate utility of individuals. There are two broad ways to do this. The first way to think about aggregate utility is in terms of *total* utility. That is, aiming to maximise the total amount of utility by adding the utils, or hedons, of each individual. Alternatively, one could think about aggregate utility in terms of *average* utility, which aims to maximise the average utils or hedons among individuals. The difficulty here is that both strands of utilitarianism seem to have unpalatable entailments, as highlighted by Parfit [32]. Very broadly, total utilitarianism seems to entail the repugnant conclusion—that for a given group of individuals, there exists an even larger group whose existence would be preferable even though their average utility is such that their lives are barely worth living [33, pp. 381–390]. Conversely, average utilitarianism seems to imply that a group comprised of two maximally happy individuals is preferable to a larger group comprised of the same two maximally happy individuals, *in addition to* 1 million other individuals, each of whom experience slightly less than the maximum amount of happiness [33, pp. 419–442].

The relevance of these difficulties regarding aggregate utility, is that while I do not make any explicit claims regarding which strand of utilitarianism is preferable, I do assume that the utilitarian aim, as far as procreation is concerned, is not to have as many children as possible whose lives are barely worth living, in order to maximise total utility.^11^

With this caveat in mind, consider the wellbeing of the prospective children. The first broad point I aim to make is that it seems far from conclusive that the prospective child would experience sufficient suffering such that their life will result in overall disutility. I want to add a caveat, however, that I am referring to prospective children that would, if brought into existence, be in relatively good health and experience a life in which their basic needs are met. Although this fails to capture a great number of human lives, it seems likely that it will capture the lives of many prospective children vegans could bring into existence. It seems to me that, on average, such lives may well include more wellbeing than suffering and therefore result in overall utility, as far as the experience of the prospective child is concerned. If brought into existence, the prospective child may go on to have a good life in which they experience deep and rewarding relationships, an enjoyable and fulfilling career, and a generally positive moment-to-moment experience.^12^ Obviously, the prospective child will almost certainly not suffer in the ways, or to the degree, that factory farmed animals do. That is, the prospective child will not have extremely poor living conditions, be mutilated, and slaughtered. It is of course a possibility that the prospective child could have a terrible life. However, the important point here is that while the vegan has good reason to believe that factory farmed animals’ lives are overwhelmingly conducive to disutility, the same is not true of the human lives I am referring to. Many human lives, possibly the vast majority of those who are in good health and who have their basic needs are met, may well experience a wellbeing to suffering ratio which results in a net utility.

Before proceeding, I want to mention that Räsänen pre-empts the move I make here and argues roughly as follows in response: Human life contains less wellbeing and more suffering than people generally realise. The pleasures one experiences in life are generally short-lived, whereas the pains are not. Examples include the pleasure that comes from having a tasty meal, juxtaposed with the pain that comes from suffering with depression. Moreover, human life contains unique ways to suffer, such as existential dread, or the pain of a long-term relationship ending, which are not experienced by factory-farmed animals [1]. Benatar [34] has provided similar considerations meant to support the notion that human lives, even the best of human lives, contain more bad things than good things. For example, chronic pain is common, whereas chronic pleasure is not, the worst pains are worse than the best pleasures are good, and it is more likely that things go wrong for people than that they go right [35, p. 76].

In response to Räsänen’s above reasoning, although there are indeed short-lived pleasures and long-lived pains, the converse is also true. That is, there are various long-term pleasures and short-term pains. Examples of long-lived pleasures could include: the satisfaction of having a fulfilling career, the contentment from knowing that one’s loved ones are safe, deep relationships, and enjoyable hobbies. All of these seem constitutive of a great number of human lives. Examples of short-lived pains can include suffering from a cold, the anxiety experienced before and during public speaking, and the minor daily inconveniences that occur at work or at home, such as running late or forgetting to run an errand. Moreover, although there are unique ways in which humans suffer, there are also unique ways in which they experience wellbeing. For example, the wellbeing humans experience when having a deep conversation with a loved one, or when appreciating art, or when being mindful of a beautiful sunset.

Moreover, even if one grants the above considerations which are intended to support anti-natalism, such as it being the case that the worst pains are worse than the best pleasures are good, and that pains are generally long-lived whereas pleasures are not, this does not entail, nor make it plausible, that the average human life involves experiencing more suffering than wellbeing. In other words, the above considerations could all be true, and it could nevertheless be the case that the average human life is, overall, characterised by wellbeing rather than suffering. For example, supposing that it is true that the worst pains are in fact worse than the best pleasures are good (which seems plausible), it may nevertheless be the case that humans experience far more pleasures than they do pains, such that the wellbeing one experiences outweighs the suffering. I of course concede that it could turn out to be the case that I am mistaken, and that the prospective human lives would actually be characterised by suffering. However, the primary point I am making here, is that the aforementioned considerations offered by Räsänen and Benatar are not sufficient to establish, nor make it plausible, that the prospective children’s lives would be characterised by suffering.^13^

In addition to drawing attention to the ways in which one suffers, anti-natalists also reference empirical literature in an attempt to show that life is worse than one thinks. For example, Benatar argues that humans are not good judges of the quality of their lives, and to that end, cites empirical research meant to support the notion that there are various ways in which humans overestimate the quality of their lives. For example, he cites literature which purports to show that humans have an optimistic bias [35, p. 67]. That is, humans overestimate the probability of experiencing positive events and underestimate the probability of experiencing negatives ones. In response to this point, it seems far from conclusive that the empirical literature, including the research Benatar cites, supports the notion that human lives are characterised by suffering rather than wellbeing, for a variety of reasons. One such reason, highlighted by Iddo Landau [35]^14^ is that although it seems true that humans may view the quality of their lives as mildly more favourable than they are, their self-evaluations regarding the quality of their lives are still quite reliable. For example, if one views the quality of one’s life as excellent, one’s life is probably just very good.^15^ Moreover, Hauskeller [36] points out that there is also evidence to the contrary, i.e., that people are happier that one might expect.^16^ Generally, it seems that the anti-natalist strategy of drawing attention to the ways in which humans suffer is not an appropriate means by which to determine what the prospective child’s experience will be overall. Furthermore, it seems that the evidence regarding the wellbeing to suffering ratio of human life is far from conclusive as far as the empirical literature is concerned. Therefore, it seems that vegans do not have good reason to conclude that the life of their prospective child will result in an overall disutility for the child.

What about the wellbeing of the prospective parents if they were to have children? The answer to this question appears to be quite complex. Literature examining the comparative wellbeing of parents and non-parents seems to indicate that there is mixed evidence regarding who is happier, with factors such as age and income playing a significant role [37]. However, as far as I can tell, it seems that non-parents are generally happier than parents. To this end, Räsänen has argued that having children is not necessary for a good life. He cites empirical research which purportedly shows that non-parents have higher life satisfaction than parents. Moreover, he argues that there are many non-parents who live very good lives [1].

In response, I want to start by acknowledging that having children is, of course, not required for the good life, or at least it is not required for everyone. However, what is of interest to this discussion, is what effect having children will have on the prospective parents in terms of utility (relative to the utility generated if they were to abstain from having children). The primary point I want to make here is that what is of relevance is not whether parents experience more or less wellbeing or suffering than non-parents. Rather, it is whether by abstaining from having children, prospective parents would experience more or less wellbeing or suffering than if they were to have children. The outcome of this latter question may differ from the outcome of the former. This is because it will often be the case that non-parents *did not desire having children* in the first place, whereas parents *did desire having children*. In other words, whether individuals are non-parents by choice, or due to undesirable circumstances, will play a significant role in the wellbeing or suffering which results from being non-parents. For those who do not want to have children (plausibly the vast majority of those who make up the “non-parent” cohorts in the relevant literature), it seems plausible that remaining childless would result in overall utility for the prospective parents. However, for those who do want children, there is not good reason to believe that by abstaining from having them, more utility will be generated in terms of their wellbeing. On the contrary, it seems plausible that if vegans who want children were to abstain from having them, as Räsänen argues they should, this would be a great source of disutility for said prospective parents.^17^

In sum, while there is good reason to believe that factory farming results in a net disutility, there is not good reason to believe that having children does. If I am correct, then vegans can accept the utilitarian argument for veganism without being committed to the utilitarian argument for anti-natalism.

## Rights-based arguments

Räsänen acknowledges that for many individuals, the motivation for being vegan is not utility-based, but rather rights-based. As such, Räsänen provides a rights-based argument for veganism and an analogous rights-based argument for anti-natalism, again arguing that vegans who accept the argument for veganism, should also accept the one for anti-natalism. His rights-based argument for veganism is:

‘P1) It is immoral to treat individuals who are subjects-of-a-life^18^ as mere means.

P2) Non-human animals are subjects-of-a-life.^19^

P3) Factory farming treats non-human animals as mere means.

C) Factory farming is immoral.’ [1, p. 144].

And here is his rights-based argument for anti-natalism:

‘P1) It is immoral to treat individuals who are subjects-of-a-life as mere means.

P2) Children are subjects-of-a-life.

P3) Having children uses children as mere means.

C) Having children is immoral.’ [1, p. 145].

In response, I am going to argue that the rights-based argument for veganism seems plausible, whereas the rights-based argument for anti-natalism does not. More precisely, while P1 and P2 of both arguments seem true, I am going to argue that P3 is true only in the argument for veganism. Firstly however, I need to clarify what is meant by “mere means”. This is tricky as there are various understandings of what qualifies as using someone as a mere means and Räsänen does not appear to provide a definition.^20^ Given the reasoning Räsänen provides, to the conclusion that children are used as mere means (discussed below), and that no definition of “mere means” has been provided, I will proceed under the assumption that Räsänen is offering a standard understanding of the term, something like: to treat someone as a mere means, is to treat them as ‘*a mere instrument or tool: someone whose well-being and moral claims we ignore, and whom we would treat in whatever ways would best achieve our aims.*’ [41, p. 213].

With this in place, I am going to start by showing my reasoning that P3 is true in the argument for veganism. This is fairly straightforward. Factory farming consists in bringing animals into existence to further human interests. That is, the goal of factory farming is to efficiently produce meat and animal products for humans to consume. Although it is true that measures which seek to minimise animal suffering are legally required in many cases, such as stunning the animals prior to slaughtering them, it is nevertheless the case that animals suffer substantially and have their most basic interests, such as being free and not being harmed or slaughtered, undermined so that humans can achieve their aims. This constitutes using the animal as a mere means to an end. The end being a desirable diet for humans.^21^

With respect to the argument for anti-natalism, Räsänen argues in support of P3, that prospective parents’ primary reason for having children is that *they want to have them* [1]. My general response to this is that one cannot infer from the proposition ‘the parents’ primary reason for having children is that they want them’, to the proposition ‘prospective parents use the prospective children as mere means by having them’. It seems extremely implausible that parents bring their children into existence to achieve their own aims *with little to no regard for the interests and wellbeing of the prospective children*. On the contrary, it seems that the interests of the children are of great importance to the parents, to the extent that it is often prioritised over the parents’ own interests, such that if there were to be a conflict between the two, the parents would choose to promote the interests of their children at the expense of their own.

Consider, for example, the dire circumstances of a parent who must choose between having a child who will suffer immensely to no good end from some congenital condition or aborting the foetus and never becoming a parent. It seems very plausible that many prospective parents in such circumstances would prioritise the interests and wellbeing of the prospective child—to not suffer immensely, over their own interests and wellbeing—to become parents. One might object here that the example I offer, intended to support the notion that the interests of the prospective child will be of great importance to the prospective parents, is problematic because the prospective child’s life is not worth living. That is, one could object that the prospective parents want to bring *a healthy child* into existence, not a child who will suffer immensely. Thus, my example is not an instance of prospective parents prioritising the interests and wellbeing of the prospective child over their own, as it is not in the parents’ interests to have a child whose life is not worth living. In other words, both the prospective parents’ and the child’s interests are for the prospective child not to suffer immensely. Thus, it could be the case that by refraining from having a child who would suffer immensely, the prospective parents are actually acting in accord with their own interests.

In response to this objection, I take it to be the case that since for many prospective parents a necessary condition for wanting a child, is that the child not suffer immensely, is evidence that the prospective parents do not use their children as mere instruments, whose wellbeing they disregard in order to achieve their own aims. Thus, I take it to be the case that prospective parents do not treat their children as mere means by bringing them into existence.

In summary, there is good reason to believe that factory farming results in treating non-human animals as mere means. Conversely, it seems implausible that having children constitutes treating said children as mere means. If I am correct, then vegans can accept the rights-based argument for veganism without being committed to the rights-based argument for anti-natalism.

## Unnecessary suffering

Before concluding, I want to respond to what I take Räsänen’s broad aim to be—to show that since vegans are motivated to boycott factory farming because it causes unnecessary suffering, that they are also committed to avoiding other practices which cause unnecessary suffering, in this case having children. I will argue that this notion is plausibly false under the operating definition of “unnecessary suffering”, i.e., the kind of suffering one could easily avoid inflicting on others, and hence that vegans are not necessarily committed to other practices merely because they also reduce avoidable suffering.^22^ The aim of this criticism is to show that vegans need not worry about being committed to a variety of other such practices.

In other words, it appears implausible that many vegans ought to avoid practices which cause unnecessary suffering given that only some of these practices will be impermissible on utilitarian or rights-based grounds. Consider, for example, the current speed limit. This is something vegans do not generally oppose (at least to my knowledge). However, the current speed limit is responsible for a certain amount of suffering, in the form of traffic accidents, which would be avoided if the speed limit were lowered. Lowering the speed limit slightly, for non-emergency vehicles, would plausibly be a fairly easy thing to do that would not come with any threat to humanity’s continued existence. Similarly, vegans do not generally oppose construction for entertainment purposes, i.e., building bars, cinemas, and theatres. Presumably, humans do not require the number of bars, cinemas, and theatres that currently exist. That is, it seems that humans could easily avoid constructing at least *some* of them.^23^ Moreover, it is known that certain people will inevitably be injured, and therefore suffer, as a result of the construction work. However, such practices seem permissible on both utilitarian and rights-based grounds. The current speed limit allows one to travel more quickly and efficiently, allowing for more time with family and friends, more sleep, and less time spent commuting. Moreover, bars, restaurants, cinemas, and theatres are beneficial to the economy and provide entertainment to billions of people.

What seems more plausible than vegans being committed to all practices which reduce unnecessary suffering (in the way Räsänen uses the term), is that vegans are committed to boycotting factory farming because of the amount of pain, interest frustration, and rights violations suffered by the animals, in exchange for the comparatively small return—one’s mere taste pleasure and convenience. Such a trade-off seems impermissible on utilitarian and rights-based grounds, as I have argued above.

## Conclusion

In this paper, I have addressed arguments which attempt to show that vegans who abstain from eating meat and animal products for moral reasons, ought to, by parity of reasoning, abstain from having children. After considering Räsänen’s utilitarian and rights-based arguments for veganism, and their counterpart arguments for anti-natalism, I have attempted to show that while the former are plausibly sound, the latter are not. As such, I have argued that vegans who accept one or both arguments for veganism, need not accept either of the arguments for anti-natalism. Finally, I have addressed the overarching theme of Räsänen’s reasoning—that since vegans want to prevent unnecessary suffering, they should not have children given both that it is not necessary and that the children will inevitably suffer throughout their lives. I have argued that vegans are not committed to abstaining from all practices which cause unnecessary suffering in the way Räsänen defines the term. The upshot of my criticism is that there is not good reason to accept the claim that vegans ought not have children.

## Footnotes

1 In this context, unnecessary suffering refers to the kind of ‘suffering that one could easily live without inflicting on anyone. For instance, if the only way to save your life is to cause you to suffer, then the suffering is not unnecessary’ [1, p. 3].

2 It is worth mentioning that the term “veganism” is not always understood in this way. The general usage of “veganism”, or “vegan”, seems to be to denote someone who abstains from eating meat and animal products. Under this definition, the motivation for being vegan is unknown. One could be vegan for an array of different reasons. For example, one could be vegan for health reasons, environmental reasons, animal welfare reasons, or merely because of taste preferences. The Vegan Society [2] uses yet another definition, which stipulates that veganism is a way of life which seeks to exclude all forms of exploitation of, and cruelty to, animals as far as is possible and practicable. Given the different understandings of veganism, the definition used here will plausibly fail to capture how many use the term. However, it is the definition that allows for proper engagement with Räsänen's arguments on his own terms.

3 Like veganism, anti-natalism has also been defined in various ways. For example, some have defined it as the view that it is preferable to never have been born, and hence that it is wrong to procreate [4], while others define anti-natalism merely as the view that it is wrong for people to reproduce [5]. This latter definition leaves open the possibility that the reason it is wrong to have children is that it would harm the prospective parents. Much like my usage of the term “veganism”, the way anti-natalism is defined in this paper will also fail to capture how some use the term. However, I will once again be using Räsänen’s definition to properly engage him on his own terms.

4 Whilst there are other forms of animal farming which cause the animals less suffering, there are utility-based reasons to avoid these farms too, albeit weaker reasons. This is because these farms also involve various practices which cause the animals unnecessary suffering in exchange for human taste pleasure. Such practices involve, at minimum, killing the animal at a young age. However, it may also involve practices such as forcibly impregnating cows and subsequently removing calves from their mothers so that the milk produced can be used for human consumption. Moreover, if the demand currently placed on factory farms were placed on these other farms instead, it seems plausible that animal welfare standards would drop to meet the high demand for animal products.

5 There are, of course, other factors that will play a role the net utility, or disutility, of having children. These may include: the prospective child’s impact on the lives of others, their carbon footprint, their career, etc. Factoring the above considerations into the utility calculus is not within the scope of this paper. This is partly due to the obvious practical limitations of accounting for all the possible variables which can affect utility. That being said, it seems plausible that the wellbeing of the prospective parents and the prospective child will play a saliant role with respect to whether it is permissible to have children on utilitarian grounds. This consideration also applies to the utilitarian argument for veganism.

6 I am using wellbeing and suffering to denote both pleasure/desire satisfaction and pain/desire frustration respectively.

7 Food and Agriculture Organization of the United Nations estimates [16]. For a breakdown of various studies over the past decade see: [17]. Alternatively, see Brown’s “7 reasons why meat is bad for the environment” [18].

8 For more, see [26, 27].

9 See Position of the American Dietetic Association [28]. See also the British Dietetic Association’s position [29].

10 I want to make it clear that there are various arguments in favour of anti-natalism that I will not be responding to here. One such example is Benatar’s asymmetry argument which stipulates that the absence of pain is good even if there is no one to experience it, whereas the absence of pleasure is only bad if there is someone who is deprived of it [3]. Addressing such arguments are out with the scope of this paper as my aim is to show vegans are not committed to anti-natalism in virtue of subscribing to veganism. For more on Benatar’s asymmetry argument, see: Benatar [3]. For some arguments against Benatar’s asymmetry argument, see: DeGrazia [30] and Bradley [31].

11 Addressing total and average utilitarianism and their criticisms is out with the scope of this paper. For further discussion, see: Parfit [32].

12 It is worth noting that others, such as Harman [34, p. 783] and DeGrazia [31, p. 164] have responded to Benatar [3] along similar lines, arguing that there is not good reason to accept that human lives are characterised by disutility.

13 It is worth noting that a life characterised by suffering is supposed to denote a life that is not worth living or starting. I do not intend to imply that all suffering is bad, as some suffering may well be constitutive of human flourishing (e.g., the suffering required to achieve worthwhile goals).

14 See especially [36 pp. 340–344].

15 For a critical breakdown of the psychological research, see Iddo [35].

16 For evidence highlighted by Hauskeller, see Myers [38].

17 The authors of the paper ‘Happy People Have Children’ [39] seem to agree that non-parents who want children, but cannot have them, would plausibly be less happy than prospective parents who want and have children.

18 In this context, being a subject-of-a-life denotes having beliefs and desires; perception, memory, feelings of pleasure and pain; preference and welfare-interests; and the ability to initiate action in pursuit of their desires and goals among other things. [1].

19 One might object here that not all non-human animals are subjects-of-a-life. For example, sea anemones are likely not sentient, and therefore not subjects-of-a-life under the operating definition. In response, although Räsänen’s argument is imprecise in stipulating that non-human animals are subjects-of-a-life, given that only a subset of them are, it is nevertheless true that the animals who are standardly factory farmed (e.g., cows, chickens, and pigs) are plausibly subjects-of-a-life in the relevant sense.

20 Definitions of treating someone as a “mere means” include end sharing – if agent 1 cannot share the ends that agent 2 is pursuing in using her, then agent 2 is treating agent 1 as a mere means. Other definitions involve possible consent and actual consent. For a thorough breakdown, see [40].

21 By ‘desirable’ I mean something like ‘taken to be healthy’, ‘tasty’, ‘convenient’, or something alike.

22 Of course, some vegans may have the ultimate goal of reducing “all” unnecessary suffering. Such vegans would be committed to these other practices. However, those who are vegan for utilitarian or rights-based reasons may in fact not have good reason to abstain from such other practices and/or may find the very idea of eliminating “all” suffering to be unrealistic or unattainable.

23 While these examples may not be great ones, my main goal here is just to highlight that there are practices which cause suffering that one could easily avoid without risk to one’s continued existence, which vegans need not be committed to abstaining from on utilitarian or rights-based grounds.
